# Phylogenomic Analysis Reveals Deep Divergence and Recombination in an Economically Important Grapevine Virus

**DOI:** 10.1371/journal.pone.0126819

**Published:** 2015-05-18

**Authors:** Hans J. Maree, Michael D. Pirie, Kristin Oosthuizen, Rachelle Bester, D. Jasper G. Rees, Johan T. Burger

**Affiliations:** 1 Agricultural Research Council, Infruitec-Nietvoorbij (The Fruit, Vine and Wine Institute), Private Bag X5026, Stellenbosch, 7599, South Africa; 2 Department of Genetics, Stellenbosch University, Private Bag X1, Matieland, 7602, South Africa; 3 Institut für Spezielle Botanik und Botanischer Garten, Johannes Gutenberg-University, Anselm-Franz-von-Bentzelweg 9a, 55099, Mainz, Germany; 4 Department of Biochemistry, Stellenbosch University, Private Bag X1, Matieland, 7602, South Africa; 5 Agricultural Research Council, Biotechnology Platform, Private Bag X5, Onderstepoort, 0110, South Africa; Kunming University of Science and Technology, CHINA

## Abstract

The evolutionary history of the exclusively grapevine (*Vitis spp*.) infecting, *grapevine leafroll-associated virus* 3 (GLRaV-3) has not been studied extensively, partly due to limited available sequence data. In this study we trace the evolutionary history of GLRaV-3, focussing on isolate GH24, a newly discovered variant. GH24 was discovered through the use of next-generation sequencing (NGS) and the whole genome sequence determined and validated with Sanger sequencing. We assembled an alignment of all 13 available whole genomes of GLRaV-3 isolates and all other publicly available GLRaV-3 sequence data. Using multiple recombination detection methods we identified a clear signal for recombination in one whole genome sequence and further evidence for recombination in two more, including GH24. We inferred phylogenetic trees and networks and estimated the ages of common ancestors of GLRaV-3 clades by means of relaxed clock models calibrated with asynchronous sampling dates. Our results generally confirm previously identified variant groups as well as two new groups (VII and VIII). Higher order groups were defined as supergroups designated A to D. Supergroup A includes variant groups I-V and supergroup B group VI and its related unclassified isolates. Supergroups C and D are less well known, including the newly identified groups VII (including isolate GH24) and VIII respectively. The inferred node ages suggest that the origins of the major groups of GLRaV-3, including isolate GH24, may have occurred prior to worldwide cultivation of grapevines, whilst the current diversity represents closely related isolates that diverged from common ancestors within the last century.

## Introduction

The history of grapevines and viticulture is intertwined with that of early human civilisations. Archaeological evidence of early viniculture can be traced back as far as the Chalcolithic and mid-Bronze Ages with evidence of vinification found in clay jars from 7000 BCE and archeobiological remains of pressed grapes from the 5th millennium BCE [1–3]. The cultivation of grapes has historically denoted high social status, and grapevines were planted wherever humans travelled and settled. Unfortunately, grapevine is susceptible to intracellular pathogens, of which many cause disorders that reduce plant vigour and longevity, as well as yield and quality of the harvest [4]. Infectious intracellular agents such as viruses, viroids, and phloem- or xylem-limited prokaryotes, of which there are more than 70 known species, are some of the most important pathogens affecting grapevine [4]. Worldwide, the most economically important viral disease is Grapevine Leafroll Disease (GLD) with *grapevine leafroll-associated virus* 3 (GLRaV-3) being the most prevalent associated virus [5]. GLRaV-3 only infects *Vitis spp*. and although it is transmitted by soft scale insects, its worldwide distribution might be attributed to the commercial trade of infected material. The origin of GLD remains vague, but it may have been in the “Old World”, predating the widespread use of phylloxera-resistant rootstocks from the USA [5].

GLRaV-3 is a single-stranded RNA virus with a genome that is organised in 12 or 13 open reading frames (ORFs). It is the type species of the genus *Ampelovirus*, family *Closteroviridae*. GLRaV-3 isolates have been classified into six groups (I-VI) according to phylogenetic analyses of the coat protein (CP) gene, of which groups I-V are more closely related and separate from group VI. Group VI represents a more diverse assemblage, serving as a convenient label for various more distantly related variants. From the limited data available for gene regions other than the coat protein, isolates classified in this group seem to lack the ORF 2 known from groups I-V and show very low levels of homology for ORFs 11 and 12 [5]. However, whether the coat protein alone can adequately represent the evolutionary history of GLRaV-3, and hence whether the current classification is likely to be predictive with regard the biological properties of these pathogens, remains an open question.

Viruses present a number of challenges for phylogenetic inference, in particular due to their high evolutionary rates (making assessment of homology more difficult) and their small genomes (restricting the total amount of potentially available data). These phenomena are also apparent in GLRaV-3 with a complete genome size of approximately 18500 nucleotides (nt), and troublesome homology assessment both within the group and in comparison to its closest known relatives. No previous analyses have included outgroups to root the GLRaV-3 phylogeny. These difficulties are compounded by the current lack of sequence information available compared to that of other important virus pathogens. There is especially a paucity of published whole genome sequences: at the time of writing, only 13. The current phylogenetic hypotheses for GLRaV-3 are largely based on the Hsp70h and CP genes and no attempts have been made to date the origin of GLRaV-3 clades.

In this paper, we set out to infer a maximally representative phylogenetic hypothesis for GLRaV-3, using as much of the currently available data as can be meaningfully assessed using phylogenetic methods, and for the first time using outgroup comparison to root the tree. We follow the phylogenetic approach of Visser et al. [6] that involves a stringent test of phylogenetic evidence for recombination and does not assume a strictly bifurcating species tree. We use asynchronous sampling of isolates to calibrate relaxed clock phylogenetic analyses. The latter allow us both to estimate the ages of internal nodes and to test the rooting of the tree. We use the results to assess the relationships within the group as a whole and in particular of a novel genetic variant of GLRaV-3, discovered using next-generation sequencing (NGS), the whole genome sequence of which is presented for the first time here.

## Materials and Methods

### Source material

Samples of *Vitis vinifera* cv. Cabernet Sauvignon vines displaying symptoms of GLD were collected from privately owned farms in the Stellenbosch region of South Africa. Explicit permission was gained from the relevant managers before sampling. Collected material was maintained in a greenhouse (Stellenbosch University, South Africa). Although vine GH24 displayed typical GLD symptoms, it only tested positive for GLRaV-3 with ELISA and all attempts to amplify GLRaV-3 from this sample via RT-PCR proved unsuccessful. Phloem scrapings and petiole material of isolate GH24 was sampled and stored at -80°C.

### Next-generation Sequencing

The use of metagenomic NGS to establish the total viral complement of a sample has been shown to circumvent the need for prior sequence information [7]. The extraction of double-stranded RNA (dsRNA) is a strategy to enrich for the replication intermediate of RNA viruses, which significantly increases the virus-specific reads in the dataset. Double-stranded RNA was extracted from GH24 phloem material using an adapted cellulose affinity chromatography method [8]. In brief: Two cycles of cellulose affinity chromatography was performed using a batch method with washing and elution steps performed in a column. Twenty grams of phloem scrapings and petioles were used for the extraction and the cellulose powder used was MN 2100 (Macherey-Nagel). The integrity and concentration of the dsRNA was assessed by gel electrophoresis (1% Agarose TAE).

An NGS library (~300nt insert) was prepared using the TruSeq RNA Sample Preparation Kit (Illumina) and sequenced in a paired-end (2x 100nt) run on an Illumina HiScanSQ at the Agricultural Research Council’s Biotechnology Platform in Pretoria, South Africa.

Paired-end sequence data was used as single reads for the *de novo* assembly of a near-complete draft sequence of GLRaV-3 isolate GH24 using CLC Genomics Workbench 6.0.2 (Qiagen). The trimmed and filtered sequence data was assembled into contigs using the following parameters: word size of 20nt, bubble size of 50nt and minimum contig length of 500nt. The quality trimmed and filtered data (5037331 reads) was submitted to the NCBI SRA database (SRR1693181). GLRaV-3-related contigs were identified using the BLAST functions, BLASTn and tBLASTx on the GenBank database. The read coverage and depth of the contigs were examined and the low coverage threshold set at 10-fold. The contigs were trimmed accordingly and aligned against a reference sequence (GLRaV-3 isolate GH30) using the BioEdit 7.0.9.0 software [9]. A draft genome sequence was compiled and used as a reference for primer design to construct the complete genome sequence of this novel variant.

### Sanger sequencing of isolate GH24

Direct Sanger sequencing of amplicons was used to confirm the sequence of the new divergent variant of GLRaV-3 and to fill the gaps in the genome sequence that were not covered by the NGS data. Primers were designed to produce overlapping amplicons, spanning the entire draft genome of the novel variant of GLRaV-3 using Oligo Explorer 1.1.0 (Gene Link), (S1 Table). Primers were also designed to amplify the 5’- and 3’-termini, in combination with an oligo(dT) primer, of a poly-adenylated dsRNA template (poly(A)-tailing) [10].

Total RNA was extracted from 2g of petioles using a modified cetyltrimethylammonium bromide (CTAB) method [11] and subjected to a two-step RT-PCR. Complementary DNA (cDNA) was synthesized from 500ng of total RNA, primed with 0.25μM gene-specific reverse primers in a reaction containing: 1X AMV Reverse Transcriptase Buffer; 10U AMV Reverse Transcriptase (Thermo Scientific) and 10mM dNTP’s in a final volume of 20μL. Individual 25μL PCR reactions were performed with 5μL cDNA containing 0.4μM of each gene-specific primer; 1X KAPA Taq Buffer A; 0.05U KAPA Taq DNA polymerase (Kapa Biosystems); 10mM dNTP’s and 10% cresol red. PCR cycling conditions included an initial denaturation step at 94°C for 5 minutes followed by 35 cycles of a denaturing step at 94°C for 30 seconds, an annealing step at the appropriate annealing temperature for 30 seconds and an extension step at 72°C for 70–105 seconds (depending on the amplicon size). The PCR was ended with a final extension at 72°C for 7 minutes. Amplicons were separated on 1% Agarose-TAE gels and fragments of expected sizes excised (Zymoclean Gel DNA Recovery Kit, Zymo Research) and directly sequenced with the designed primers.

To determine the 5’- and 3’ -termini of the genome, poly(A)-tailing was performed on dsRNA with yeast poly(A) polymerase (Affymetrix) followed by cDNA synthesis using an oligo(dT) primer [10]. Genome-specific reverse (for the 5’-terminus) and forward (for the 3’-terminus) primers were designed (S1 Table) and used in combination with the oligo(dT) primer during PCR amplification. Amplicons of expected sizes were cloned (pGEM-T Easy Vector, Promega) and a minimum of five clones for each terminus, sequenced with T7 and SP6 primers.

All sequences were trimmed to remove low quality bases. The vector sequences were also removed from the cloned amplicon data. Using BioEdit, the trimmed sequences were aligned to the draft sequence and a complete genome sequence was compiled. To resolve ambiguities, CLC Genomics Workbench was used to map the NGS data to the Sanger sequencing-generated reference genome. All ambiguities were replaced with the nucleotide that was most abundant at that position in the NGS data.

Open reading frames were predicted using NCBI ORF Finder (http://www.ncbi.nlm.nih.gov/gorf/gorf.html), and domains were predicted by the Pfam 27.0 software [12]. The whole genome sequence of GLRaV-3 isolate GH24 was deposited in GenBank (KM058745).

### Sequence alignment and supermatrix construction

To infer the phylogeny of GLRaV-3, and in particular to assess the phylogenetic position of the new variant of GLRaV-3, we constructed a supermatrix that included a total of 819 GLRaV-3 accessions, represented by sequences of differing lengths, obtained from GenBank. At the time of the analysis, only 13 complete GLRaV-3 genome sequences were available. In our analysis we regarded isolate NY-1 and CL-766 as complete since all ORFs of these isolates are complete and the genomes only lack a portion of the 5’ UTR. Genome regions that are more commonly sequenced are ORF 4 (Hsp70h) with 135 sequences and ORF 6 (CP) with 576 sequences. The 3’region of the genome spanning ORF 6 to 12 is represented by 50 sequences contributed by a single study [13]. All sequences were aligned using ClustalW followed by manual adjustment using amino acid translation of codon positions as represented graphically in Mesquite [14] in order to minimise apparent shifts in reading frame that would imply loss of function. The positions of ORFs in the alignment were defined as character sets, using isolate GP18 as a reference [15]. A number of ORFs are known to overlap: ORFs 4 and 5 (8nt), ORFs 8 and 9 (4nt) and ORFs 11 and 12 (4nt). The overlapping portions were treated as a separate (putatively highly conserved) further character set. *Grapevine leafroll-associated virus* 1 (GLRaV-1) was selected as the outgroup. Due to the differences in genome organization between GLRaV-1 and GLRaV-3, only the GLRaV-1 ORFs corresponding to those of GLRaV-3 were included in the alignment. The supermatrix with ORF partitions is available in nexus format (S1 File). Using this alignment, pairwise comparisons of only the 13 complete GLRaV-3 genomes were performed with CLC Main Workbench 6.8.3 to determine percentage nucleotide identities between isolate GH24 and the other genomes. Nucleotide and amino acid identities for all the ORFs were also compared. Synonymous and non-synonymous substitutions were assessed for each ORF individually with MEGA6 [16] using the Kumar model [17] with whole genomes only, excluding the outgroup, and with all positions containing gaps and missing data eliminated.

### Recombination detection and phylogenetic analysis

Multiple recombination detection methods as implemented in RDP4 [18] were used to identify putative recombination breakpoints. The methods included RDP [19], GENECONV [20], MaxChi [21], Bootscan/Recscan [22], SiScan [23] and 3Seq [24]. Default settings were used and the threshold *p*-value set at 0.05, using Bonferroni correction. Following Visser et al. [6], these potential breakpoints were tested using phylogenetic analyses of the corresponding putatively non-recombinant regions under parsimony (using PAUP* [25], estimating clade support using 1,000 replicates of bootstrap analysis, each replicate involving heuristic search settings of 100 random taxon additions with tree bisection and reconnection branch swapping, saving a maximum of 100 trees each) and Maximum Likelihood (ML; using RAxML [26] on CiPRES [27], with heuristic search followed by fast bootstrapping halted automatically by RAxML following the majority-rule ‘autoMRE’ criterion). The resulting sequence of trees represented a first estimate of the stepwise changes in phylogenetic signal resulting from recombination. For each accurately inferred breakpoint a single topological conflict was expected; when this was apparent and subject to bootstrap support (BS) of ≥70% (under both methods), the corresponding breakpoint was accepted; when not, it was rejected; and where multiple topological differences between successive trees were supported (implying failure to identify additional breakpoints), the breakpoints in this region of the alignment were reassessed and the process repeated. The end result is a conservative estimate of recombination between sequences including identification of recombinant isolates. It will fail to identify more recent recombination events and/or those involving short sequence regions, but in these cases the corresponding sequence variation is low and it is likely to have little influence on subsequent phylogenetic analyses [6].

Having identified recombination breakpoints and the corresponding recombinant isolates, it was possible to modify the supermatrix for phylogenetic analyses without violating the assumption of an underlying bifurcating tree. This was achieved by constructing an alignment using the ‘taxon duplication’ approach [28,29], whereby the recombinant sequences are split into multiple taxa, each taxon representing one phylogenetically conflicting gene region with the rest of the alignment re-coded as missing data. Multiple recombinants were treated following Visser et al. [6], excluding non-contiguous recombinant regions of the genome where a common phylogenetic signal could not be identified with confidence. This approach avoids the pitfalls of combining conflicting phylogenetic signals, irrespective of the cause of such conflict.

PartitionFinder [30] was used to test the fit of combinations of data partitions (assuming the GTR+G substitution model implemented in RAxML, i.e. without a parameter for the proportion of invariant sites (I) and without removing such sites prior to analysis) given a matrix of whole genome sequences only with recombinants excluded, thus avoiding potential influence of missing data. We applied a heuristic search strategy (‘greedy’) and comparison of fit by means of the Bayesian information criteria. Potential data partitions were each of the codon positions of each of the 13 ORFs individually (39 partitions), plus the regions of overlapping ORFs (highly conserved), and non-coding regions (unconserved), making a total of 41. Following these results, partitioned ML analyses were performed on a matrix of whole genome sequences only with putative recombinants treated as multiple taxa. The resulting multi-labelled tree was summarized as a rooted network using the HOLM 2006 algorithm in Dendroscope [31,32].

In order to infer a phylogenetic hypothesis that was both maximally representative (in terms of isolates) and resolved (in terms of supported nodes, particularly for the major groupings of GLRaV-3), analyses (RAxML; PAUP*) were performed that included increasing numbers of isolates that were represented in the supermatrix with decreasing lengths of sequence data. Of 819 GLRaV-3 accessions, 498 were represented by at least 504nt; 392 by ≥602nt, 374 by ≥942nt; 69 by ≥1658nt; 65 by ≥4761nt; and 14 (including the outgroup) by ≥19362nt.

To estimate node ages (i.e. the ages of common ancestors of different GLRaV-3 strains) and to make an estimation of the rooting of the GLRaV-3 tree that is independent of comparison to the (genetically rather distant) outgroup, we performed Bayesian phylogenetic inference and molecular dating using BEAST with the outgroup removed [33]. The analyses were restricted to include only isolates for which a meaningful isolation date could be obtained (either from the literature or by personal communication with the authors). The spread of tip ages used for age calibration was rather narrow (between 2003 and 2011), with many isolates originating from the same year (2009). Following preliminary analyses that indicated that convergence was problematic with missing data and more complex models, we included only the 64 ingroup taxa represented by ≥4761nt and only the corresponding 4761nt of sequence alignment that was complete for these taxa; the data were not partitioned: a single GTR+G with estimated base frequencies substitution model was applied. A likelihood ratio test was performed using PAUP* with this dataset and an arbitrary shortest tree, comparing the fit of GTR+G models with and without enforcing a strict molecular clock. The strict clock model was rejected (P>0.999), and two different relaxed clock models applied, assuming a) lognormal and b) exponential rate distributions. A coalescence demographic model was applied assuming constant population size. Two independent runs of 200 million generations, sampling every 20000 were performed for each relaxed clock model.

## Results

### Next-generation sequencing of isolate GH24

NGS data was bioinformatically analysed to determine the viral composition and the possible cause of GLD in this plant. *De novo* assembly of NGS data yielded 316 contigs of which 33 were identified to be of viral origin similar to GLRaV-3, and marginally closer related to variants of group VI. On average these contigs shared 73.69% sequence identity with isolate GP18 (group II) and 74.26% with isolates GH11 and GH30. These contigs were trimmed and aligned to isolate GH30 to compile a draft sequence of the novel variant, isolate GH24. The genome was estimated to be 84.71% complete and consisted of 18647nt with the largest gap being 1319nt long.

### Sanger sequencing of isolate GH24

Direct sequencing of RT-PCR amplicons was used to construct approximately 98% of the GLRaV-3 isolate GH24 genome, spanning all of the gaps in the draft sequence and validating the NGS *de novo* assembly. All sequences overlapped between 38-142nt and were aligned to the draft sequence that was covered fully except for approximately 60nt on either terminus. Twelve ambiguities were observed in the Sanger sequence data that were temporarily retained and resolved later by mapping the NGS data to this new sequence. The identity of each base was determined by the most frequent occurring nucleotide in the NGS read data. By resolving the ambiguities, the *in silico* characterization of the complete genome, such as predicting ORFs and conservative domains, was made possible.

The 5’-terminal nucleotide was found to be a cytosine. This additional cytosine residue at the start of the genome sequence is the complement of the additional guanine residue at the 3’-terminus of the negative strand of the virus. The extra residue was first observed in *citrus tristeza virus* (CTV) and is described as a non-template residue that may reflect the presence of a cap structure at the 5’-terminus of the RNA virus [34]. Having amplified the negative strand of the dsRNA to determine the 5’-terminal nucleotide, the residue was excluded when compiling the consensus sequence.

The complete genome of GLRaV-3 isolate GH24 was found to be 18493nt long with average sequence similarities to other GLRaV-3 whole genomes ranging between 63.39 and 65.11%. These similarities are low compared to values of >85% observed between isolates of group I, group II, and group III. The similarity to other whole genomes also varied across the genome. Additionally, GH24 shows high sequence similarity (99%) to partial genome sequences of isolate CB19 collected in the USA (EF445655-702nt and EF445656-602nt), isolate Tempr collected in Italy (DQ314610-1807nt), and isolate GTG10 collected in South Africa (KC731553-549nt and KC731554-517nt). Isolate GH24 was found to exhibit a typical GLRaV-3 ORF 1a domain structure and genome organization, however, no ORF 2 could be identified.

Through multiple pairwise comparisons the nucleotide and amino acid identities of the various ORFs and UTRs were compared between isolate GH24 and the other GLRaV-3 isolates. The 5’UTR of isolate GH24 was found to be 737nt in length, similar to those of isolates 621, WA-MR, 3138–07, GP18, 623, GH11 and CA7246, but with low sequence similarity. The intergenic region between ORF 1b and 3 was observed to be 6nt shorter than that of isolate GP18 with low sequence similarity, ranging between 37.94 and 52.15% (S2 and S3 Tables). The 3’UTR is more conserved and consists of 259nt. Open reading frame 9 terminates one amino acid earlier than ORFs 9 of other GLRaV-3 isolates. ORF 12 has a premature stop codon and is four amino acids shorter than that of group I, II and III isolates. Results indicate that ORFs 9 to 12 are less than 65% identical at amino acid level compared to other isolates of GLRaV-3. ORF 11 shares less than 30% amino acid identity to that of isolates of groups I to III, and approximately 46% to that of group VI isolates. Overall, the ORF nucleotide identities range between 39.64 and 78.75%, whereas the percentage identity at amino acid level is shown to range between 21.62 and 90.76%. Average overall synonymous (Ks) and non-synonymous (Ka) substitutions and the Ka/Ks ratio for ORFs 1a and 1b, and ORFs 3–12 are presented in Table 1. Ka/Ks ratios were <1 in all but ORF1b, 5, and 12. Comparisons within-groups was incalculable in: ORF8 for group VI vs VII; ORF10 for group I vs VI; and ORF11 for group VII vs all other groups and group VII vs I, II.

**Table 1:** Synonymous (Ks) and non-synonymous (Ka) substitutions.

ORF	Ks	Ka	Ka/Ks
1a	0.937	0.132	0.141
1b	0.042	0.214	**5.095**
2	0.506	0.418	0.826
3	0.658	0.083	0.126
4	0.815	0.06	0.074
5	0.1	0.312	**3.12**
6	0.885	0.036	0.041
7	0.84	0.106	0.126
8	0.696	0.089	0.128
9	0.69	0.237	0.343
10	0.857	0.184	0.215
11	0.317	0.213	0.672
12	0.299	0.434	**1.452**

Average overall Ks and Ka substitutions and the Ka/Ks ratio for each ORF as assessed with MEGA6 using the Kimura model. Values for Ka/Ks >1 indicated in bold type represent evidence for positive selection.

### Phylogenetic analysis

**Fig 1:**
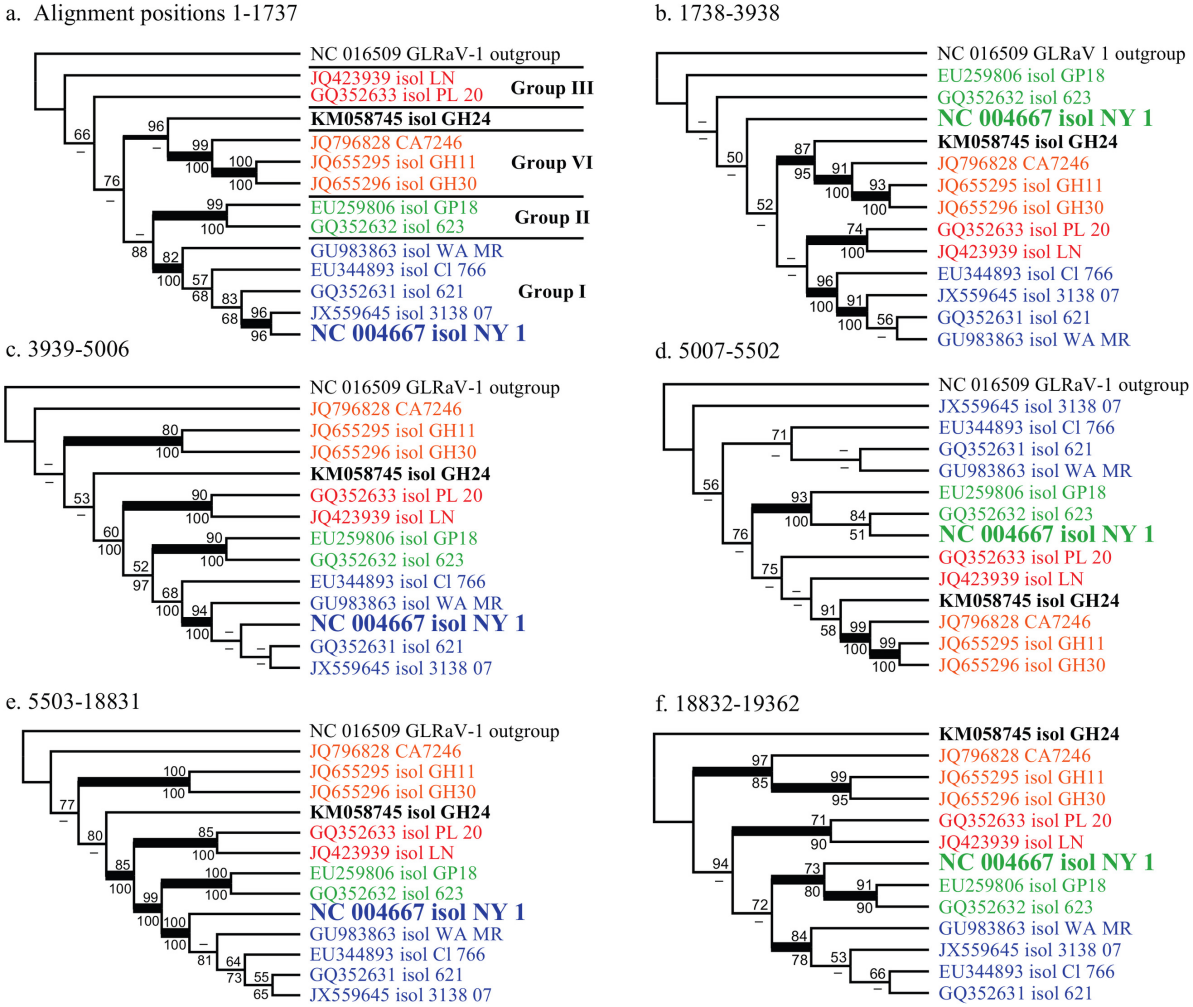
Maximum likelihood phylogenetic analysis of six putatively non-recombinant gene regions. Alignment positions are indicated. Isolates are coloured according to group, as indicated, and NY-1 (NC 004667), subject to topological conflicts between trees is indicated with larger font.

Preliminary results from RDP4 analyses indicated eight isolates as putatively non-recombinant, with seven putative recombination events detected amongst the other five isolates. Three of the putative recombination events were not reflected in topological conflict given phylogenetic analyses of non-recombinant regions. The corresponding breakpoints were therefore rejected. Supported conflicts were limited to isolate NY-1 (NC_004667), which was a multiple recombinant as indicated by multiple changes in phylogenetic signal across the genome (Fig 1). Evidence for recombination breakpoints in isolate WA_MR (GU983863) at multiple positions (3050, 7000, 13549 and 14382), representing topological conflict within the group I clade, received significant but not high support under ML (BS up to c. 80%), but <70% BS under parsimony (data not shown). Topological conflict in isolate GH24 was moderately supported under both methods, but the results were inconsistent: GH24 is sister to group VI under both methods between positions 1 and c. 7000 (Fig 1a–1d), after which there is a shift to sister to groups I, II and III supported under ML (80% BS; Fig 1e); but a shift to sister to the rest of GLRaV-3 was supported under parsimony (84% BS; not shown). In both cases the corresponding breakpoints were rejected.

**Fig 2:**
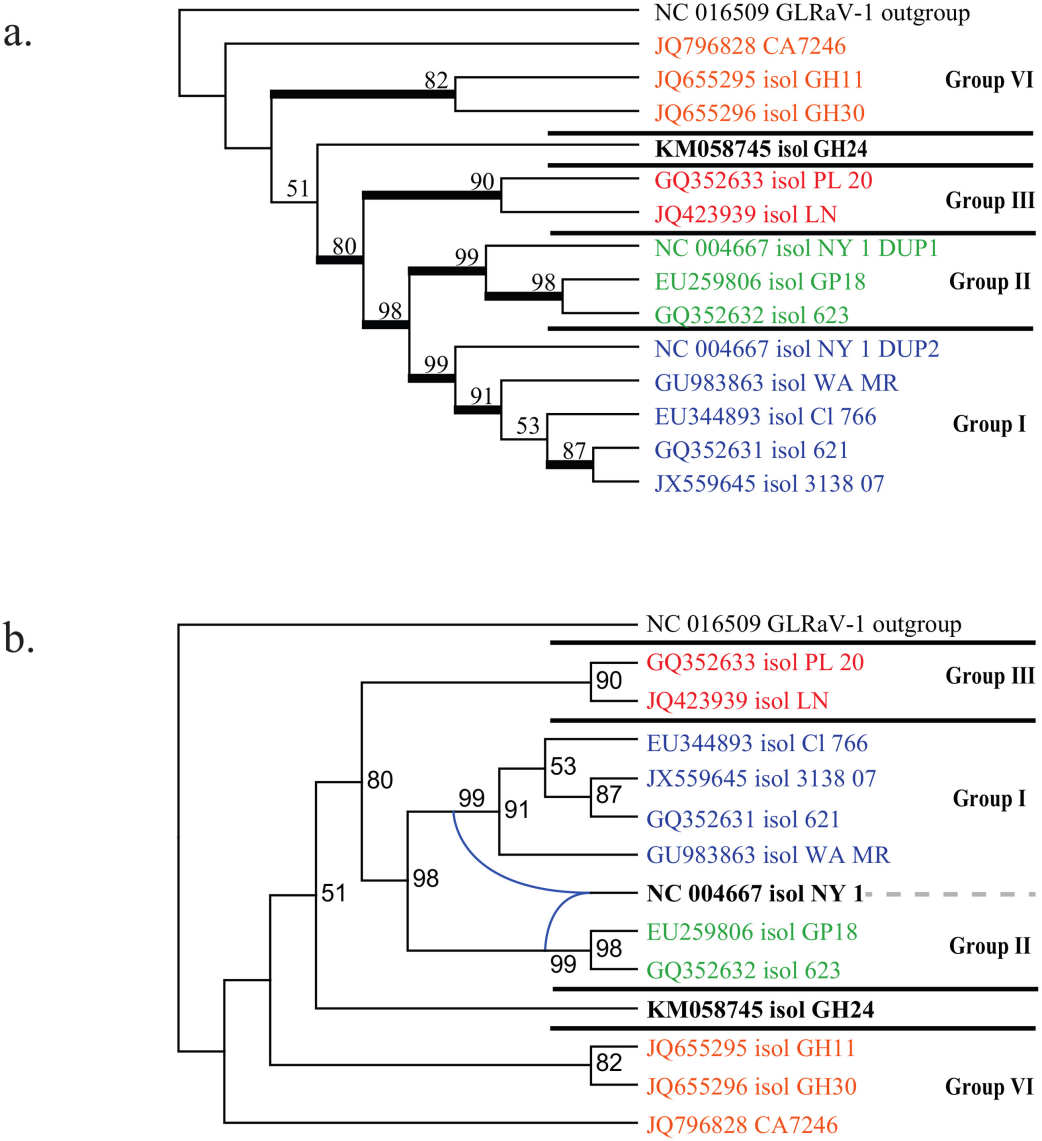
Phylogenetic analysis of the whole genomes under partitioned ML. (a) Multi-labelled ‘genome’ tree and (b) the corresponding rooted network inferred using Dendroscope.

For downstream analysis the recombinant sequence was represented by two separate taxa, one for each of the non-recombinant regions, excluding shorter recombinant regions. The best fitting partitioning strategy inferred using PartitionFinder included 8 independent substitution models. Across different ORFs, the same positions in the triplet code were frequently grouped under the same linked substitution model. Phylogenetic analysis of the whole genomes using the taxon duplication approach resulted in a multi-labelled ‘genome’ tree (Fig 2a). Resolution and support were greater under parsimony than under ML, including bootstrap support (BS) of 100% for monophyly of Group VI, and 85% BS for monophyly of groups I-III and VI to the exclusion of GH24 (neither supported under ML). The ML tree is also presented as a rooted network inferred using Dendroscope (Fig 2b).

**Fig 3:**
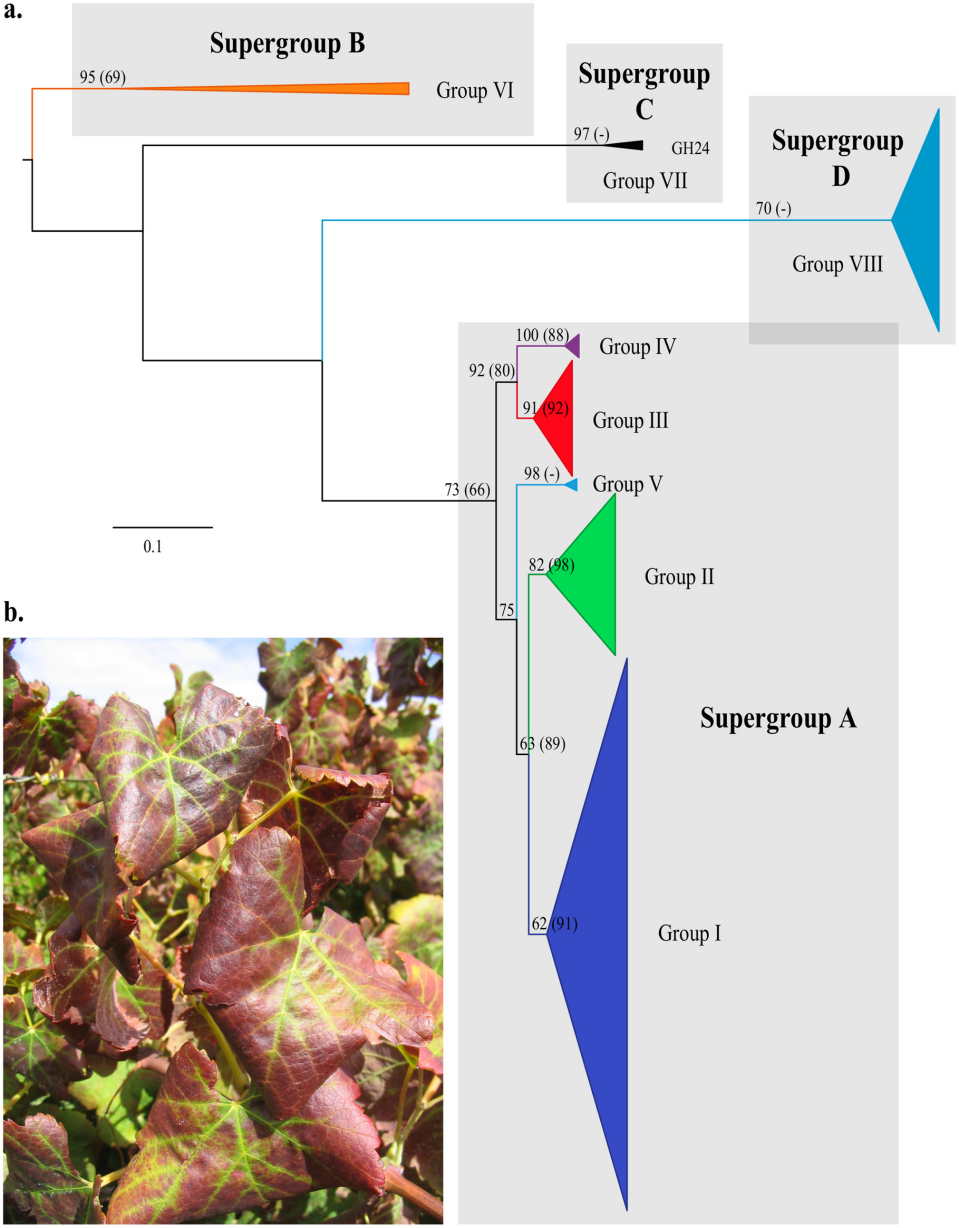
Summary of RAxML phylogenetic analysis. (a) 392 GLRaV-3 sequences of 602nt or more in length was used for the analysis. The tree is rooted, but the outgroups have been removed for ease of presentation; the scale indicates branchlengths in substitutions per site. Values at nodes are ML bootstrap support: first, given this tree, thereafter (within parentheses) support for the equivalent clade given the analysis including sequences of 4761nt or more in length. Groups and supergroups proposed here are indicated. Further details, including support values and tip labels are presented in supporting information S2 Fig. (b) A grapevine (*Vitis vinifera* cv. Cabernet Sauvignon) showing typical symptoms of Grapevine leafroll disease.

Results of phylogenetic analysis of the supermatrix including 392 sequences of 602nt or more in length are summarised in Fig 3, and presented in detail along with trees based on taxa with minimum sequence length of 504nt, 602nt, 942nt, 1658nt and 4761nt in supporting information S1–S6 Figs Excluding isolates with sequences shorter than 602nt resulted in overall improved clade support (particularly for the major groupings), compared to the ≥602nt tree. Excluding isolates with sequences shorter than 942nt resulted in an apparent change in the character polarisation compared to the ≥602nt tree, resulting in a basal grade of short or zero-length branches. In Fig 3, currently recognised variants are indicated, as well as two clades representing new variant groups VII and VIII. Four more inclusive clades are also indicated, defined as supergroups A to D. Supergroup A includes variant groups I-V; supergroup B variant group VI and isolate NZ2; supergroup C includes variant group VII (isolate GH24) and supergroup D, variant group VIII. Accession EF103904 is sister to the GLRaV-3 clade; it is treated as an outgroup (see Discussion) and not represented in Fig 3. Pairwise comparison of EF103904 to the whole genome sequence of isolate 139 (GLRaV-3m, JX266782) revealed 99% similarity. The basal nodes, representing the order of divergence between GLRaV-3 supergroups, and hence the rooting of the tree, were not supported.

**Fig 4:**
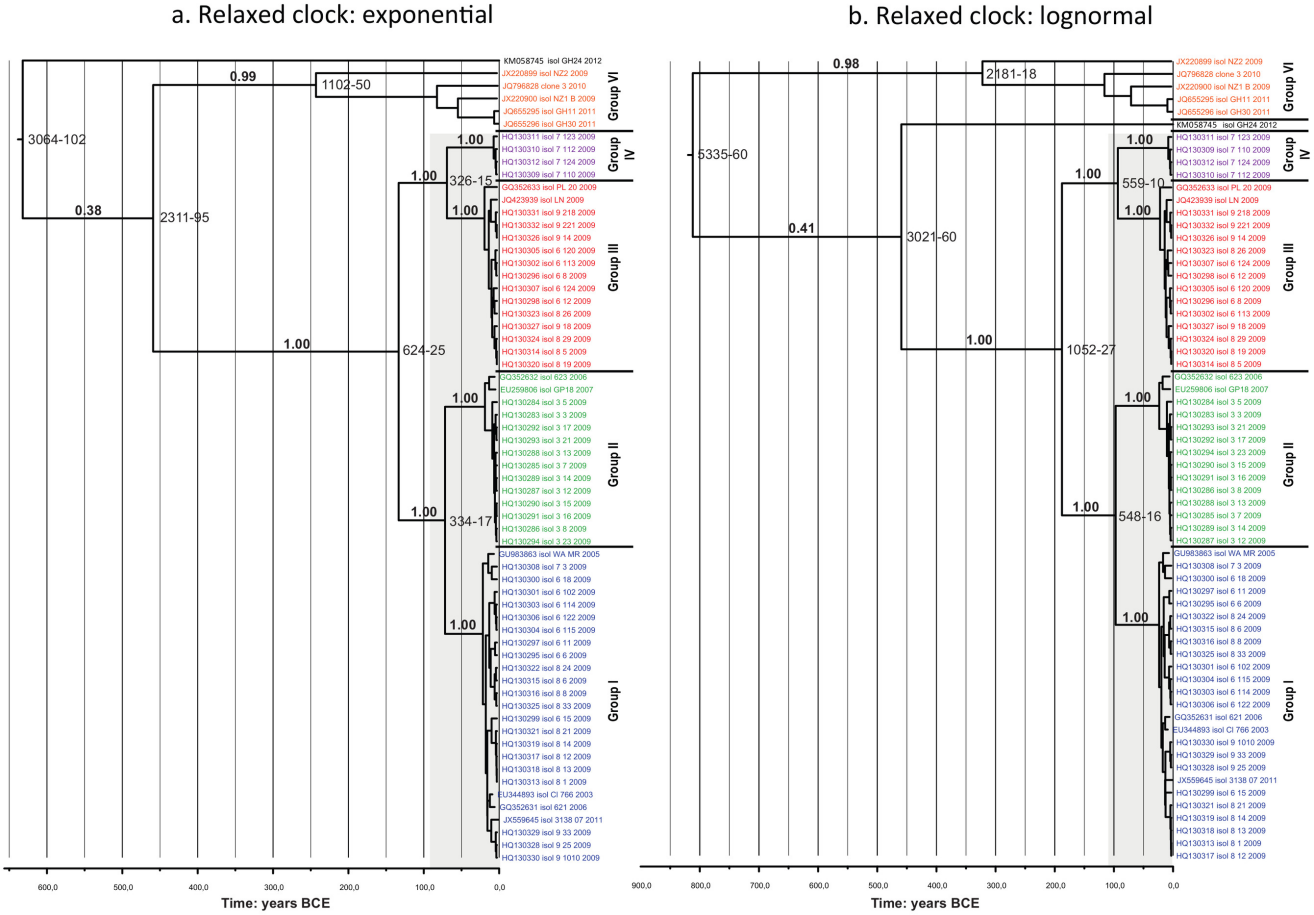
Bayesian phylogenetic inference and molecular dating analysis. BEAST was used for estimation of node ages and rooting of the GLRaV-3 tree, independent of the outgroup. a. Exponential relaxed clock. b. Lognormal relaxed clock. Variant groups are indicated and colour coded following Figs 1–3. For the major clades, posterior probability (PP) clade support is indicated above the branches; 0.95 PP age ranges at the nodes. The extent of 0.95 PP age ranges for nodes within groups I-IV is indicated by grey boxes.

Sixty-four isolates, for which sampling date and at least 4761nt of sequence data were available, were used to infer a time-calibrated tree using BEAST (Fig 4). The rooting of the tree, estimated without the outgroup, is subject to uncertainty similar to that apparent in the results of outgroup-rooted analyses based on the complete genomes. The topology is otherwise consistent, albeit in the absence of representatives of groups V and VIII, for which sequences of comparable length were not available. Posterior probability distributions of molecular rates (in mutations per site per year), given the two different relaxed clock models, were: LN: mean 7.4487E-4 (95% highest posterior density (HPD) = 8.5454E-8—1.8636E-3); EX: mean 9.4624E-4 (95% HPD = 3.7556E-5—2-0539E-3). Posterior probability distributions of node ages indicated low precision for deeper nodes in general and of the divergence of supergroup B in particular, but the latter appears to be relatively ancient, as is the divergence of isolate GH24. Crown group ages for groups I-IV were rather more precise, with the 0.95 PP ranges falling within the last century.

## Discussion

In this study we assessed the evolutionary history of GLRaV-3, the type strain of the genus *Ampelovirus*, and determined the phylogenetic placement of a new genetic variant, isolate GH24 (KM058745).

### A first ‘genome tree’ of *grapevine leafroll-associated virus 3*

We included sequences of as many GLRaV-3 isolates as possible in our analyses. To this end, we constructed a supermatrix of 819 accessions, including the 13 whole genomes, with the larger part of the alignment comprising of missing data. It has been shown in both empirical and simulation studies that the proportion of missing data in a matrix does not in itself significantly impact phylogenetic analysis [35]. Instead, the important factor is the presence of informative sites necessary to place particular taxa. Most previous studies of GLRaV-3 have concentrated on the RNA-dependent RNA polymerase (RdRp), the coat protein (CP) and the heat shock protein 70 homologue gene (Hsp70h) [5,36–42], which for most known isolates, are the only sequence data currently available. The generally low clade support in the phylogenetic trees that we inferred when including GLRaV-3 isolate sequences shorter than c. 600nt suggest that more than half of the currently available sequences (427 of the 819) are insufficiently informative to be placed with precision in a phylogeny of the group. Nevertheless, relatively short sequences i.e. of c. 600-900nt, including ORF6, can be placed with confidence when analysed within our supermatrix including full genome and other longer sequences.

Next-generation sequencing technologies should yield several increasing numbers of whole genomes in the near future, which will enable more full-length sequence comparisons and necessitate a reassessment of recombination between them. Currently, only one of the 13 full genomes analysed to date showed a clear mosaic of phylogenetic signals, while some evidence for further such patterns exist in another two. Multiple infections could potentially lead to similar results, but our analytical approach is not sensitive to the underlying cause of the phylogenetic conflict. Assuming that these sequences do indeed represent single infections, and not lab artefacts, these results are indicative of past recombination. Such recombination has not been detected in previous work, probably because the putative recombination breakpoints fall outside the largest body of available data, i.e. functionally more conserved CP and Hsp70h.

In the construction of our GLRaV-3 ‘genome tree’, GLRaV-1 was used as the outgroup, even though it is genetically very distant. This presents a frequently encountered problem in phylogenetic analyses: it is difficult to infer the polarisation of character state changes given a highly divergent group with a long stem lineage. Uncertainty in the precise phylogenetic position of GH24 and the somewhat inconsistent results comparing ML with parsimony analyses of non-recombinant gene regions is probably symptomatic of this uncertainty in the rooting. There are also apparent changes in character polarisation in the ML trees (S1–S6 Figs) and presumably spurious short or zero length terminal branches at the base of the ingroup, involving either clone WC-HSP-10, isolates of supergroup D, or GH24, depending on the sampling but irrespective of sampling dates. This uncertainty is further reflected in the BEAST analyses which resulted in ultrametric trees that are, by definition, rooted, without the need to specify an outgroup, but which differed between the two relaxed clock models that we applied (Fig 4). The best way to address this issue in future work will be to add further sequences that are more similar to GH24, and ideally to identify further, preferably less divergent, outgroups. Clone WC-HSP-10 (EF103904), although representing a short and somewhat enigmatic sequence, may be the most appropriate candidate for a less divergent outgroup for GLRaV-3. As a divergent sister group to GLRaV-3 in these analyses, this isolate and the very similar GLRaV-3m isolate 139 (JX266782) require further investigation as potential further members of GLRaV-3.

### A novel genetic variant and classification of *grapevine leafroll-associated virus 3*

Our results validated the discovery of a new highly divergent variant of GLRaV-3, isolate GH24. For the first time the complete genome of this variant was sequenced. As well as allowing us to infer its distant phylogenetic relatedness to other known strains, the results showed that isolate GH24 shares less than 66% sequence identity, at nucleotide level, with all GLRaV-3 isolates readily attributable to known strains. GH24 does show high sequence similarity to partial genome sequences of isolates collected in the USA, Italy and South Africa. These isolates are likely representatives of the same genetic variant, suggesting that this variant already occurs across the world.

Comparison of the overall non-synonymous and synonymous substitution ratios indicated that ORF1b, 5 and 12 are under positive selection while the rest of the genome appears to be mostly under purifying selection. The within-group comparisons for ORF8, 10 and 11 were incalculable for some variants that might have influenced the ratio. The analysis will benefit from the addition of more whole genome sequences to establish the overall trends.

Our phylogenetic analyses were performed under different methods with contrasting assumptions, but delivered similar results. These results are generally consistent with trees previously inferred on the basis of CP alone, and hence with the corresponding current classification (groups I-VI). The putatively recombinant isolate that we identified, NC 004667 isol NY-1, indicated recombination between strains of different groups (I and II), but these are closely related. We consider it useful to recognise within GLRaV-3 two new variant groups designated group VII, including isolate GH24 and related isolates, and group VIII. These eight GLRaV-3 variant groups can be grouped into four supergroups. Groups I to V together represent a clearly monophyletic group in our analyses (Fig 4) that we refer to as supergroup A. Group VI variants show greater genetic diversity than those in supergroup A, but are also clearly monophyletic and share genome organisation characteristics that differ from isolates in supergroup A, including the lack of ORF 2 and high divergence in ORFs 11 and 12. We refer to this as supergroup B. Supergroups C and D are currently represented by the least amount of data, but even on the basis of partial genome sequences it is clear that they are both monophyletic and genetically distinct from the other supergroups to a comparable degree. Supergroup C comprises variant group VII with partial sequences from across the world with isolate GH24 being the only whole genome known for this group. Supergoup D includes only variant group VIII that consists of a collection of CP sequences that were directly submitted to GenBank, and obtained from surveys of Portuguese vines conducted in 2007, 2009 and 2010.

The relationships between supergroups are sensitive to both the region of the genome analysed and the method used to infer relatedness (i.e., assuming parsimony, maximum likelihood, or different likelihood-based relaxed-clock models). The absence of ORF 2 in isolate GH24 (supergroup C) could be interpreted to suggest that it is more closely related to supergroup B isolates, as also indicated in some of the trees of non-recombinant regions of the alignment (Fig 1e). The sequence variation observed for ORFs 11 and 12 indicates that their function might not be conserved in GLRaV-3, similar to isolate NZ2 [43], isolates GH11 and GH30 [36], as well as isolate CA7246 (JQ796828) [44]. However, from the sequence variation it is apparent that GH24 is not closely related to any other GLRaV-3 clade and on that basis can best be regarded as representing a separate supergroup.

The significant genetic distance between isolates in supergroup B and between GLRaV-3 supergroups in general, as well as the position of accessions EF103904 (clone WC-HSP-10) and JX266782 (GLRaV-3m) raises some questions about the taxonomic boundaries of GLRaV-3 as a virus species. At the nucleotide level, it might be argued that a number of additional grapevine leafroll-associated virus species could be identified. However, we would suggest that group VI, group VI-related isolates (such as isolate NZ2), and the newly defined groups VII and VIII be referred to as GLRaV-3 viruses with the supergroup as the strain identifier. This is in line with general trends in grapevine leafroll-associated virus taxonomy, in which related viral strains tend to be regarded within more broadly defined species. For example, GLRaV-4 was redefined to include GLRaV-5, GLRaV-6, GLRaV-9, GLRaV-Pr and GLRaV-Car [45]. Furthermore, little is known about the biological properties of different GLRaV-3 genetic variants. It would be important to investigate whether there is significant variation in their pathogenicity before considering delimiting further species, irrespective of the degree of sequence divergence involved.

### The age of diversification in *grapevine leafroll-associated virus 3*

A number of studies of viral pathogens of crop plants have shown diversification coinciding with the spread of agriculture, i.e. on timescales measured in thousands or even just hundreds of years [6,46]. The ease of spread of pathogens across the globe in the last century has also been cited as a cause for recombination between viral strains, sometimes resulting in an escalation of pathogenicity [6].

Molecular dating by means of serial sampling of virus isolates is a potentially powerful tool for inferring the timing of recent evolutionary processes in the absence of a fossil record [47,48]. The age estimations produced here were unavoidably imprecise given the small number of sequences and their recent ages that were included in the dating analyses. Nevertheless, the confidence intervals around diversifications within the major groups of GLRaV-3 were narrow and also recent. The dating analysis indicated that the lineage of supergroup C (including isolate GH24) pre-dates diversifications of supergroups A and B. Supergroup D was not included in the dating analysis due to a lack of data.

Our results would be consistent with a scenario in which the major groups of GLRaV-3 originated prior to worldwide cultivation of grapevines, whilst the current diversity represents closely related isolates that diverged from common ancestors within the last century. If this is true, we might expect the greatest genetic diversity of GLRaV-3 to be found in the native ranges of the host plants. However, since the movement of grapevines, and presumably their associated pathogens, has been ongoing throughout recorded history, it is possible that much of the diversity of GLRaV-3, such as strains similar to GH24, is to be found worldwide.

## Supporting Information

S1 Fig RAxML phylogenetic analysis of the 498 GLRaV-3 sequences of ≥504nt. The best scoring tree with branch lengths representing substitutions/site and bootstrap support for nodes above the branches; presented using TRED http://www.reelab.net/tred/default/index.
(PDF)

S2 Fig RAxML phylogenetic analysis of the 392 GLRaV-3 sequences of ≥602nt. Used to generate the cartoon in Fig 3a. The best scoring tree with branch lengths representing substitutions/site and bootstrap support for nodes above the branches; presented using TRED http://www.reelab.net/tred/default/index.
(PDF)

S3 Fig RAxML phylogenetic analysis of the 374 GLRaV-3 sequences of ≥942nt or more in length. The best scoring tree with branch lengths representing substitutions/site and bootstrap support for nodes above the branches; presented using TRED http://www.reelab.net/tred/default/index.
(PDF)

S4 Fig RAxML phylogenetic analysis of the 69 GLRaV-3 sequences of ≥1658nt or more in length. The best scoring tree with branch lengths representing substitutions/site and bootstrap support for nodes above the branches; presented using TRED http://www.reelab.net/tred/default/index.
(PDF)

S5 Fig RAxML phylogenetic analysis of the 65 GLRaV-3 sequences of ≥4761nt or more in length. The best scoring tree with branch lengths representing substitutions/site and bootstrap support for nodes above the branches; presented using TRED http://www.reelab.net/tred/default/index.
(PDF)

S6 Fig RAxML phylogenetic analysis of the 13 GLRaV-3 sequences of ≥13851nt or more in length. The best scoring tree with bootstrap support for nodes above the branches; presented using TRED http://www.reelab.net/tred/default/index.
(PDF)

S1 File Supermatrix alignment of GLRaV-3 sequences. Alignment of 819 accessions of GLRaV-3 with GLRaV-1 (NC_016509) as the outgroup
(ZIP)

S1 Table Primers used to generate isolate GH24 amplicons. (DOCX)

S2 Table Pairwise nucleotide sequence comparisons between isolate GH24 and other GLRaV-3 complete genome sequences. (DOCX)

S3 Table Pairwise amino acid sequence comparisons between isolate GH24 and other GLRaV-3 complete genome sequences. (DOCX)
