# Supplementary material for: Phylogenomic Analysis Reveals Deep Divergence and Recombination in an Economically Important Grapevine Virus
Source: PLoS One. 2015 May 18;10(5):e0126819. doi: 10.1371/journal.pone.0126819 (PMC4436351; doi:10.1371/journal.pone.0126819)
Supplement: S1 Table — (DOCX) [file pone.0126819.s008.docx]

**S1 Table. Primers used to generate isolate GH24 amplicons.**

| **Primer name** | **No. of bases** | **Primer sequence** | **Amplicon** | **Annealing** |
| --- | --- | --- | --- | --- |
|  |  |  | **size (nt)** | **temperature (°C)** |
| **Genome Sequencing** | | | | |
| LR3_GH24_11_F | 20 | TAGTAGGTATCGAACACAGC | 1 138 | 50 |
| LR3_GH24_1148_R | 20 | GGGTGTCGTCCTATAATAAT | 1 138 | 50 |
| LR3_GH24_986_F | 20 | CTTAGTTAGCGGCAATGGTG | 1 120 | 55 |
| LR3_GH24_2105_R | 20 | ACACTTGTGCGATGGTTTCT | 1 120 | 55 |
| LR3_GH24_1922_F | 18 | GATTTTGGCTTCTGTGGC | 1 466 | 55 |
| LR3_GH24_3387_R | 18 | TGACAAGCACCGACCCTG | 1 466 | 55 |
| LR3_GH24_3144_F | 20 | GCGATCATGGCTCAAGCTAT | 1 337 | 55 |
| LR3_GH24_4480_R | 20 | TGTGGTACAGATTCGTGCAT | 1 337 | 55 |
| LR3_GH24_4291_F | 20 | TCTTCGGATTGGGGACACTT | 1 018 | 55 |
| LR3_GH24_5308_R | 20 | TAGCTCTTTATTCGCCTTGC | 1 018 | 55 |
| LR3_GH24_5110_F | 18 | CGTCTCGGCACTACCTGA | 1 349 | 55 |
| LR3_GH24_6458_R | 18 | TGGCATCGTGGAAAACTA | 1 349 | 55 |
| LR3_GH24_6239_F | 20 | CCGAATCAGGTGTACGAATC | 1 319 | 55 |
| LR3_GH24_7557_R | 20 | GGCAACCATCAAGTAAATCC | 1 319 | 55 |
| LR3_GH24_7375_F | 20 | CGATGTAACAACGCAGTCTG | 1 308 | 55 |
| LR3_GH24_8682_R | 20 | ACAAACCGTCATCCACCTGG | 1 308 | 55 |
| LR3_GH24_8481_F | 18 | ACTTTGCTGTCCGTTGTA | 1 167 | 50 |
| LR3_GH24_9647_R | 18 | ATCAGCATGTGCGACTTA | 1 167 | 50 |
| LR3_GH24_9438_F | 20 | TCGTAGCGTCAGGGTAGGTT | 1 822 | 52 |
| LR3_GH24_11259_R | 20 | AGTTGTAATCAGCGGGCACC | 1 822 | 52 |
| LR3_GH24_11140_F | 20 | CACTTCTGAGGGTTGTTGAT | 1 086 | 52 |
| LR3_GH24_12225_R | 20 | CGTTCACGACTTCCTCTCCG | 1 086 | 52 |
| LR3_GH24_12055_F | 20 | TCGTCTACGGCATCTTTGAA | 1 486 | 55 |
| LR3_GH24_13540_R | 20 | TAGCGTTGGTCCCATAGATA | 1 486 | 55 |
| LR3_GH24_13334_F | 20 | AATCATTCGACCCTTTTCAT | 1 266 | 52 |
| LR3_GH24_14599_R | 20 | ATCGTCGGTTTCCACATAAT | 1 266 | 52 |
| LR3_GH24_14377_F | 19 | CGTTACCCACAAGCCAAAG | 1 443 | 55 |
| LR3_GH24_15819_R | 20 | TGATATGTCCGATGTTAGCG | 1 443 | 55 |
| LR3_GH24_15616_F | 19 | TTCGTCTCTATTCAAGGCA | 1 154 | 52 |
| LR3_GH24_16769_R | 19 | CTCACTGATATTCCGCACA | 1 154 | 52 |
| LR3_GH24_16523_F | 20 | AACCTACGATCTTCACTATG | 1 204 | 50 |
| LR3_GH24_17726_R | 19 | TGGTGACGAAACTATTGAC | 1 204 | 50 |
| LR3_GH24_17496_F | 20 | AAGGGAACGTGATTGAATAT | 1 143 | 52 |
| LR3_GH24_18638_R | 19 | TCGTCGATAAGTTAGCCTC | 1 143 | 52 |
| **Poly(A)-tailing** | | | | |
| LR3_GH24_250_R | 20 | ACTTAACACGACGCTACTCC |  | 55 |
| LR3_GH24_18273_F | 20 | AAGTGATGTGTGCGATGGTT |  | 55 |
|  |  |  |  |  |

Primer list used to generate amplicons to sequence the complete genome of the novel variant of GLRaV-3, isolate GH24.
