# Supplementary material for: Phylogenomic Analysis Reveals Deep Divergence and Recombination in an Economically Important Grapevine Virus
Source: PLoS One. 2015 May 18;10(5):e0126819. doi: 10.1371/journal.pone.0126819 (PMC4436351; doi:10.1371/journal.pone.0126819)
Supplement: S2 Table — (DOCX) [file pone.0126819.s009.docx]

**S3 Table.** **Pairwise nucleotide sequence comparisons between isolate GH24 and other GLRaV-3 complete genome sequences.**

| **GH24** | | **621** | **WA-MR** | **Cl-766** | **3138-07** | **NY-1** | **GP18** | **623** | **PL-20** | **LN** | **GH11** | **GH30** | **CA7246** |
| --- | --- | --- | --- | --- | --- | --- | --- | --- | --- | --- | --- | --- | --- |
| UTR or  ORF | Genome Position | % Nucleotide sequence identity to isolate GH24 | | | | | | | | | | | |
| 5' UTR | 1-737 | 46.34 | 46.61 | 39.24^b^ | 46.21 | 39.24^b^ | 43.34 | 43.21 | 42.54 | 46.20 | 46.16 | 43.94 | 42.16 |
| ORF1a | 738-7451 | 64.58 | 64.62 | 64.42 | 64.49 | 64.65 | 64.46 | 64.61 | 64.59 | 64.49 | 66.50 | 66.56 | 65.89 |
| ORF1b | 7393-9072 | 78.21 | 78.15 | 77.68 | 78.10 | 78.15 | 77.86 | 78.21 | 78.51 | 78.75 | 78.15 | 78.04 | 78.33 |
| UTR^a^ | 9073-10508 | 51.78 | 52.15 | 51.91 | 52.09 | 51.91 | 51.72 | 52.15 | 51.72 | 51.78 | 38.42 | 38.37 | 37.94 |
| ORF3 | 10509-10646 | 70.29 | 71.01 | 70.29 | 69.57 | 69.57 | 69.57 | 69.57 | 71.74 | 71.74 | 69.57 | 69.57 | 66.67 |
| ORF4 | 10665-12314 | 75.52 | 75.64 | 75.52 | 75.70 | 75.27 | 76.06 | 76.42 | 74.85 | 74.61 | 73.09 | 73.27 | 72.42 |
| ORF5 | 12307-13758 | 66.74 | 66.80 | 66.60 | 66.80 | 66.53 | 67.42 | 67.70 | 67.77 | 67.56 | 67.29 | 67.56 | 68.53 |
| ORF6 | 13848-14789 | 76.01 | 76.22 | 75.90 | 76.11 | 75.80 | 74.73 | 74.73 | 75.58 | 75.80 | 77.49 | 77.07 | 76.54 |
| ORF7 | 14852-16285 | 67.43 | 67.29 | 67.29 | 67.36 | 67.22 | 67.43 | 67.22 | 67.92 | 68.06 | 69.46 | 69.39 | 69.32 |
| ORF8 | 16296-16853 | 68.10 | 67.92 | 67.92 | 68.10 | 68.10 | 67.38 | 67.56 | 66.31 | 66.31 | 65.59 | 65.41 | 64.87 |
| ORF9 | 16850-17380 | 58.57 | 58.00 | 58.00 | 58.57 | 58.57 | 57.44 | 57.44 | 57.82 | 57.63 | 55.93 | 55.74 | 55.74 |
| ORF10 | 17390-17929 | 58.89 | 58.89 | 59.07 | 59.26 | 58.52 | 59.44 | 59.81 | 60.37 | 60.19 | 59.07 | 58.89 | 57.04 |
| ORF11 | 17929-18039 | 40.54 | 39.64 | 41.44 | 41.44 | 41.44 | 41.44 | 42.34 | 43.24 | 44.14 | 60.36 | 60.36 | 56.76 |
| ORF12 | 18064-18234 | 64.33 | 64.33 | 64.33 | 64.33 | 64.33 | 63.16 | 63.16 | 61.99 | 63.16 | 57.89 | 57.89 | 56.73 |
| 3' UTR | 18235-18493 | 66.89 | 67.24 | 67.58 | 67.58 | 67.24 | 67.58 | 67.58 | 68.94 | 68.60 | 75.19 | 75.19 | 73.31 |

Each ORF and UTR of isolate GH24 and that of the other GLRaV-3 complete genome sequences available on the GenBank database are compared. Nucleotide identities are given as a percentage. ^a^Group VI-like isolates lack ORF 2, therefore the UTR between ORF 1b and 3 was compared across the isolates. ^b^Isolate Cl-766 and NY-1 do not have a fully sequenced 5’UTR, therefore a partial sequence was used for the comparison.
