# Supplementary material for: Phylogenomic Analysis Reveals Deep Divergence and Recombination in an Economically Important Grapevine Virus
Source: PLoS One. 2015 May 18;10(5):e0126819. doi: 10.1371/journal.pone.0126819 (PMC4436351; doi:10.1371/journal.pone.0126819)
Supplement: S3 Table — (DOCX) [file pone.0126819.s010.docx]

**S4 Table.** **Pairwise amino acid sequence comparisons between isolate GH24 and other GLRaV-3 complete genome sequences.**

| **GH24** | | **621** | **WA-MR** | **Cl-766** | **3138-07** | **NY-1** | **GP18** | **623** | **PL-20** | **LN** | **GH11** | **GH30** | **CA7246** |
| --- | --- | --- | --- | --- | --- | --- | --- | --- | --- | --- | --- | --- | --- |
| UTR or  ORF | Genome Position | % Amino acid sequence identity to isolate GH24 | | | | | | | | | | | |
| ORF1a | 738-7451 | 68.43 | 68.48 | 68.04 | 68.39 | 67.73 | 68.17 | 68.34 | 68.26 | 68.08 | 70.36 | 70.49 | 69.92 |
| ORF1b | 7393-9072 | 88.04 | 87.86 | 86.96 | 87.86 | 87.86 | 86.43 | 86.96 | 87.86 | 87.86 | 89.29 | 89.11 | 90.00 |
| ORF3 | 10509-10646 | 71.74 | 71.74 | 71.74 | 71.74 | 71.74 | 73.91 | 73.91 | 73.91 | 73.91 | 73.91 | 73.91 | 71.74 |
| ORF4 | 10665-12314 | 86.36 | 86.55 | 86.00 | 86.55 | 85.64 | 86.18 | 87.09 | 87.27 | 86.91 | 83.64 | 84.00 | 83.45 |
| ORF5 | 12307-13758 | 74.59 | 74.38 | 74.38 | 74.38 | 74.38 | 74.59 | 75.21 | 74.38 | 74.38 | 71.69 | 72.73 | 72.52 |
| ORF6 | 13848-14789 | 90.76 | 90.76 | 90.76 | 90.76 | 90.13 | 88.54 | 89.17 | 90.13 | 90.45 | 90.76 | 89.81 | 90.13 |
| ORF7 | 14852-16285 | 75.52 | 74.69 | 75.52 | 75.73 | 75.10 | 75.10 | 75.31 | 75.10 | 75.31 | 73.85 | 73.64 | 74.27 |
| ORF8 | 16296-16853 | 70.43 | 69.89 | 69.35 | 70.43 | 70.43 | 70.43 | 70.43 | 68.28 | 68.28 | 66.13 | 66.13 | 67.74 |
| ORF9 | 16850-17380 | 53.11 | 53.11 | 52.54 | 53.67 | 53.11 | 51.98 | 51.98 | 51.98 | 51.98 | 44.07 | 43.50 | 45.20 |
| ORF10 | 17390-17929 | 51.67 | 51.67 | 51.11 | 51.11 | 51.11 | 52.22 | 52.78 | 52.78 | 52.78 | 51.11 | 50.00 | 49.44 |
| ORF11 | 17929-18039 | 27.03 | 21.62 | 27.03 | 27.03 | 27.03 | 24.32 | 24.32 | 27.03 | 29.73 | 45.95 | 45.95 | 45.95 |
| ORF12 | 18064-18234 | 63.16 | 61.40 | 63.16 | 63.16 | 64.91 | 64.91 | 63.16 | 63.16 | 63.16 | 52.63 | 52.63 | 56.14 |

Each ORF of isolate GH24 and that of the other GLRaV-3 complete genome sequences available on the GenBank database are compared. Amino acid identities are given as a percentage.
