# Supplementary material for: Phylogenomic Analysis Reveals Deep Divergence and Recombination in an Economically Important Grapevine Virus
Source: PLoS One. 2015 May 18;10(5):e0126819. doi: 10.1371/journal.pone.0126819 (PMC4436351; doi:10.1371/journal.pone.0126819)

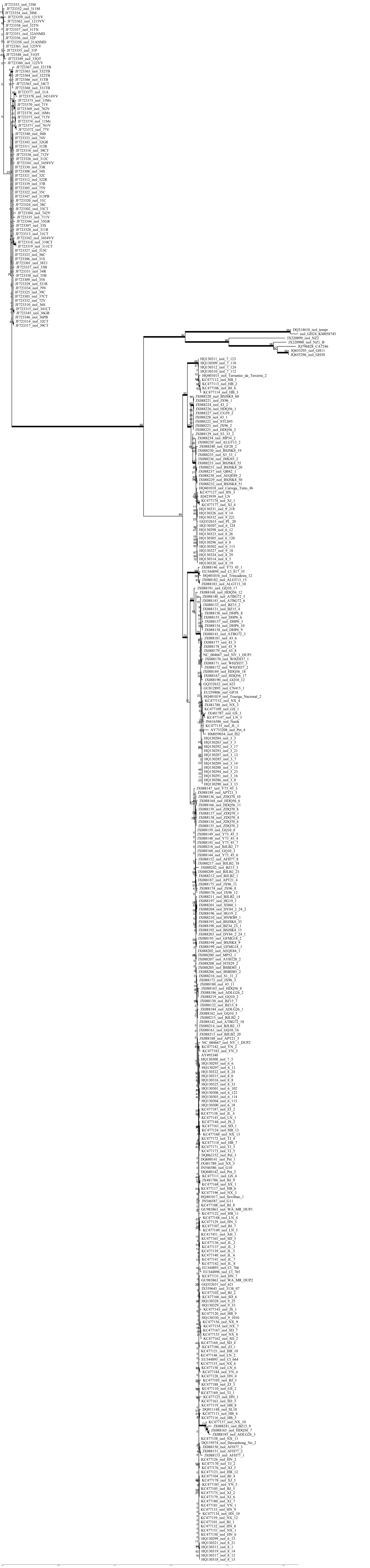

Supplement: S3 Fig — The best scoring tree with branch lengths representing substitutions/site and bootstrap support for nodes above the branches; presented using TRED http://www.reelab.net/tred/default/index. (PDF) [file pone.0126819.s003.pdf]
